# A novel index for measuring the impact of devices on hypertension

**DOI:** 10.1038/s41598-023-39943-4

**Published:** 2023-08-22

**Authors:** D. B. Kingsmore, B. Edgar, M. Rostron, C. Delles, A. J. B. Brady

**Affiliations:** 1 School of Cardiovascular & Metabolic Health, University of Glasgow, Glasgow, UK; 2 Department of Vascular Surgery, Elizabeth University Hospital, Glasgow, Queen UK; 3 Glasgow Renal and Transplant Unit, Queen Elizabeth University Hospital, 1345 Govan Rd, Glasgow, G51 4TF UK

**Keywords:** Renovascular hypertension, Renal artery stenosis, Outcomes research

## Abstract

A key limitation in assessing the therapeutic impact of non-pharmacological approaches to treating hypertension is the method of reporting outcomes. Reducing the medications required to achieve the same blood pressure may be reported separately to a reduction in the blood pressure without change in medication, and thus lessen the reported beneficial impact of treatment. This study aims to derive a novel scoring system to gauge the therapeutic impact of non-drug treatment of hypertension by utilising a combination of excessive blood pressure and the number of anti-hypertensives into a combined score—the hypertensive index (HTi). The hypertensive index was empirically derived based on the systolic blood pressure and number of antihypertensive drugs, and applied retrospectively to a cohort undergoing intervention for renovascular hypertension. Subgroup and receiver operating characteristic analyses were used to compare the HTi to traditional methods of reporting outcomes. Following intervention (99 patients), 46% had improvement in both medication load and blood pressure, 29% had benefit in blood pressure without reduction in medication load, 15% had reduction in medication load without significant change in blood pressure and 9% showed no benefit in either parameter. The HTi was superior in detecting benefit from intervention compared with measuring blood pressure or medication load alone (AUC *0.94* vs *0.85;0.84*). The hypertensive index may be a more sensitive marker of treatment effect than assessing blood pressure measurements alone. The use of such scoring systems in future trial design may allow more accurate reporting of the effects of interventions for hypertension.

## Introduction

Hypertension is the most important single modifiable risk factor for many cardiovascular diseases such as ischaemic heart disease and stroke, in addition to contributing to other long-term sequelae including end organ damage, chronic kidney disease (CKD), cognitive impairment/dementia and mortality^1–3^. The magnitude of this impact is demonstrated in a recent large-scale analysis of randomised trials that found a 5 mm Hg reduction of systolic blood pressure reduced the risk of major cardiovascular events by about 10%^4^.

Hypertension that is resistant to treatment occurs in 10–20% of patients. Partly this may relate to poor compliance with up to ½ of newly diagnosed hypertensives stopping their antihypertensive medications by 6 months, and up to one quarter of patients not taking any of their antihypertensive medications. This poor adherence to prescription may be attributable to various factors including polypharmacy, adverse effects, and the difficulty in accepting lifelong treatment for an asymptomatic disease. Therefore it is unsurprising that there is interest in non-drug treatments for hypertension^5,6^.

There are several differing approaches to devices to treat hypertension including renal artery stenting, renal denervation, vagal nerve stimulation, carotid body ablation, and arteriovenous fistula formation^5^. However, a key limitation in assessing the therapeutic impact of a device is the method of reporting outcomes, as a beneficial impact may be measured either in terms of absolute blood pressure change or by the impact on the number of drugs required. Reducing the number of medications to achieve the same blood pressure may be reported separately to a reduction in the systolic blood pressure with no change in medication, and thus lessen the reported beneficial impact of the treatment. This problem was seen in the ROX CONTROL trial, that randomised 83 patients to either standard care or insertion of AV coupler^7^. They found that whilst the device led to a reduction in blood pressure of 27/20 mmHg, 25% of patients in the coupler group decreased their antihypertensives whilst 30% of the control group had an increase in medications. Thus, intention to treat analysis of the blood pressure changes alone may have masked the true extent of beneficial effect.

There have been few previous attempts to describe ‘treated blood pressure’ in the literature. One recent example, the Genome-Wide Association Study on blood pressure and Hypertension (GWAS), used a composite measure by supplementing the systolic blood pressure by 10 mmHg if a patient was prescribed any number of antihypertensive drugs^8^. However this pragmatic approach fails to accommodate for a difference between those requiring single-agent or multi-agent treatment to maintain a specific blood pressure. In addition, the indiscriminate use of a set blood pressure equivalent of 10 mmHg is arbitrary, with few single agent randomised controlled trials achieving this measure of reduction, and an unknown efficacy of multi-drug regimens of varying type^9^.

The aim of this study was to derive a novel scoring system to gauge the therapeutic impact of non-drug treatment of hypertension by utilising a combination of excessive blood pressure (> 120 mmHg) and the number of anti-hypertensives into a combined score—the hypertensive index (HTi).

## Materials and methods

### Study population and clinical data

A retrospective analysis was performed of all patients who had received non-pharmacological intervention (renal artery angioplasty with or without stenting, unilateral nephrectomy) for the treatment of renovascular hypertension in a defined geographical area in the West of Scotland (population 2.4 million) across 3 centres between 2010 and 2022.

A database of patients with mechanical intervention performed for hypertension was used and augmented by a word-search of the radiology information system in which all imaging and interventions are reported. Case-notes were reviewed, and data obtained.

Data extracted included patient demographics as well as the indication for intervention and the systolic and diastolic blood pressure measurements before intervention and at the point of discharge following intervention. The number of prescribed antihypertensive medications was recorded at each of these time periods. 2 patients who died in the immediate perioperative period were excluded from this analysis.

### Previously published data

For comparative analysis, published data from two multi-centre studies of renal artery stenting were accessed^10,11^. Measurements relating to blood pressure and medication load before and after intervention in each study were collected to facilitate calculation of the HTi in each study cohort.

#### The Hypertensive Index

A novel marker of excessive blood pressure and medication load was empirically derived based on the systolic blood pressure and number of antihypertensive drugs.

$${\text{Hypertensive}}\;{\text{Index}} = \left( {{\text{Systolic}}\;{\text{Blood}}\;{\text{Pressure}} - {12}0} \right) \times \left( {{\text{No}}.\;{\text{Antihypertensives}} + {1}} \right)$$

The number of antihypertensive medications was supplemented by a factor of 1 to allow calculation of the hypertensive index in those requiring no antihypertensive medications.

A subgroup of patients who did not achieve significant blood pressure change was then assessed using the Hypertensive Index to evaluate for a more sensitive measure of treatment effect.

### Comparison with other measures

Receiver operator characteristic (ROC) analysis was used to compare the HTi against conventional measurements (systolic or diastolic blood pressure alone; medication load alone).

Various iterations of the HTi formula were tested to assess changes in the sensitivity and specificity depending on the blood pressure measurement component; including mean arterial pressure, diastolic pressure and pulse pressure.

To compare the Hypertensive Index against the GWAS method, pre- and post-intervention scores were calculated for each patient and the reduction in these scores compared. Scores pertaining to the GWAS method were calculated by the addition of 10 mmHg to the patients’ systolic blood pressure if they were prescribed any antihypertensive drug. ROC analysis was used to compare the efficacy of each in determining whether intervention had been ‘successful’.

Further analysis was performed to assess the potential utility of the pre-intervention HTi in predicting the impact of treatment. The published data from two multi-centre studies of renal artery stenting were analysed^10,11^. Mean pre- and post-stenting HTi scores calculated for each cohort, and their outcomes compared.

### Statistical analysis

‘Significant change’ in blood pressure was defined as a reduction in systolic blood pressure of ≥ 20 mmHg, or diastolic blood pressure of ≥ 10 mmHg^12^*.*

For the purposes of ROC analysis, ‘successful intervention’ was defined by a significant change in blood pressure without additional antihypertensive medications, or by any reduction in medication load without an increase in blood pressure.

Data was collated using Microsoft Excel (Version 16.53 © Microsoft 2021). Statistical analysis was performed using RStudio (Version 1.4.1717© 2009–2021 RStudio, PBC). Data distribution was assessed using Shapiro-Wilks Test. Means were compared by Paired T-test, Welch Two-Sample T-test or Mann–Whitney U Test as appropriate.

### Ethics

All participants provided written informed consent and all study procedures were in concordance with the Declaration of Helsinki, 1975 (revised 2000). Formal ethical approval was not required as per national legislation produced by the Health Research Authority^13^.

## Results

### Demographics of cohort

99 patients were treated for hypertension with an intervention in the time-period studied, with a median age of 63 years (IQR 53–74). Only 5 patients were non-white, an equal number were males and females, and the underlying aetiology was atherosclerotic renal artery stenosis (ARAS) in 86%. The interventions performed included RA angioplasty (n = 16), RA stent (n = 78) and unilateral nephrectomy in 5 cases for renal artery occlusive disease not amenable to endovascular treatment. 4 additional patients in whom it was not possible to fully revascularize or remove an ischaemic kidney were analysed separately.

### Impact of treatment

**Figure 1:**
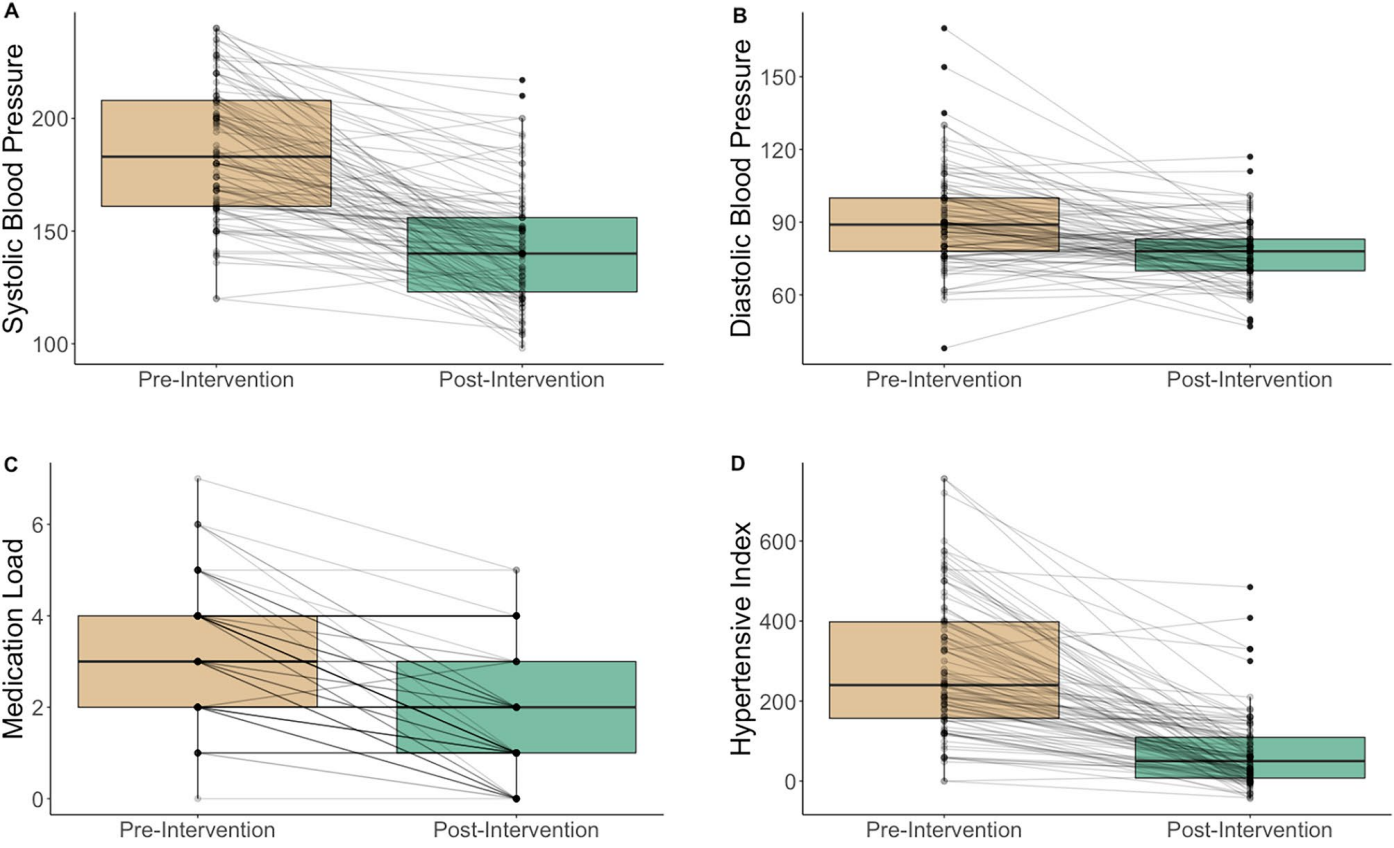
Boxplot and line graph of the response to renal artery intervention at discharge. Key: (**A**) systolic blood pressure (**B**) diastolic blood pressure (**C**) number of antihypertensive medications (**D**) hypertensive Index.

Renal artery intervention across the entire group led to a substantial reduction in systolic and diastolic blood pressure and prescribed antihypertensive medications at the point of discharge. Blood pressure fell from 185/91 to 142/77 mmHg, plus a reduction in drug therapy from 3.2 medications to 1.8 (Table 1, Fig. 1).

**Table 1:** Blood pressure and medication values before and after renal artery intervention.

	Baseline		Discharge		p-value
	Mean	95% ci	Mean	95% ci	
Systolic blood pressure	185.3	29.8	142.7	29.8	< 0.0001
Diastolic blood pressure	91.1	19.8	77.3	19.8	< 0.0001
Antihypertensive drugs	3.2	1.4	1.8	1.4	< 0.0001
Hypertensive index	283	171	70	70	< 0.0001

*ci* confidence interval.

**Figure 2:**
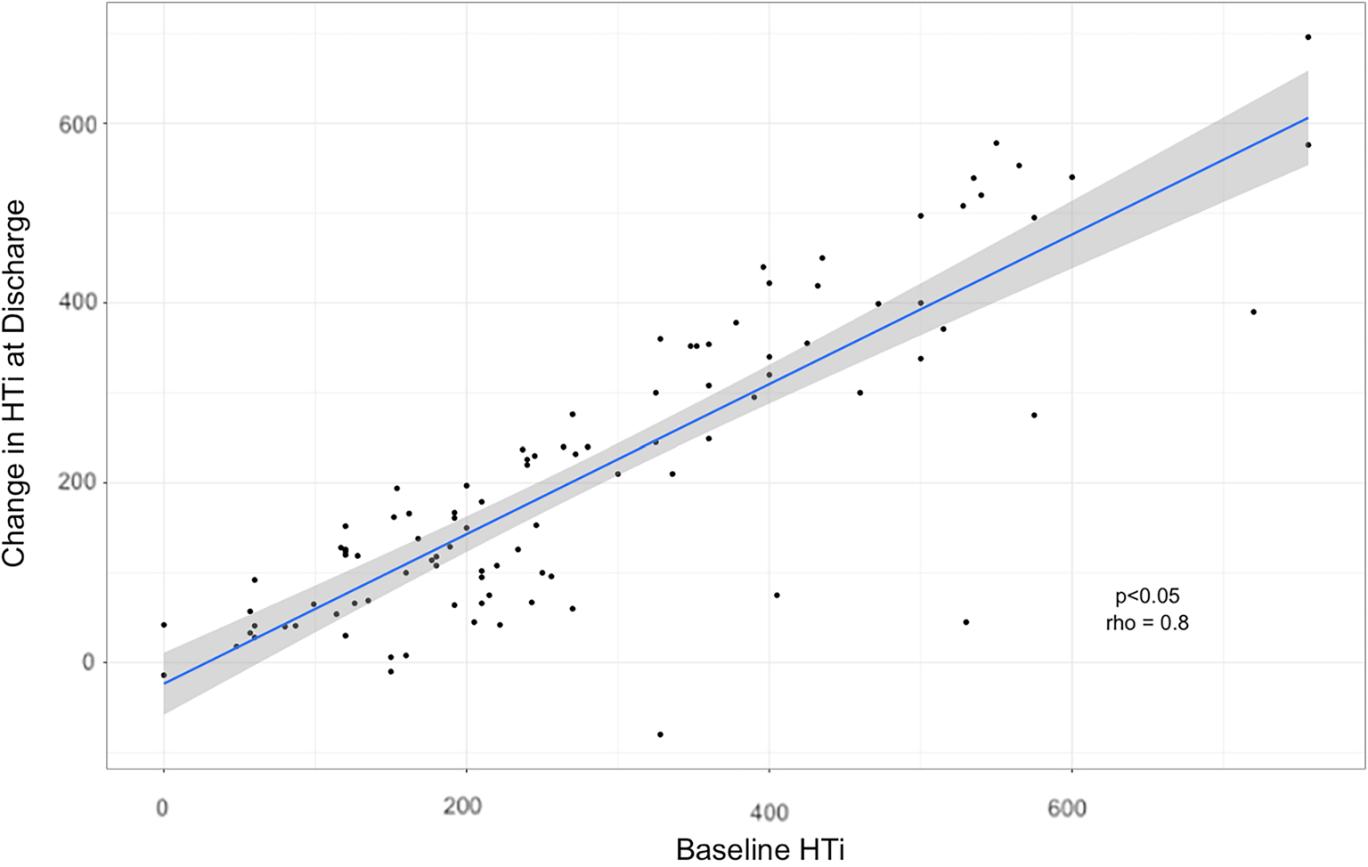
Scatter graph of pre-intervention hypertensive index and the magnitude of change following intervention. Key: HTi = Hypertensive Index. Spearman’s rank correlation coefficient (rho) = 0.8, p > 0.05.

**Figure 3:**
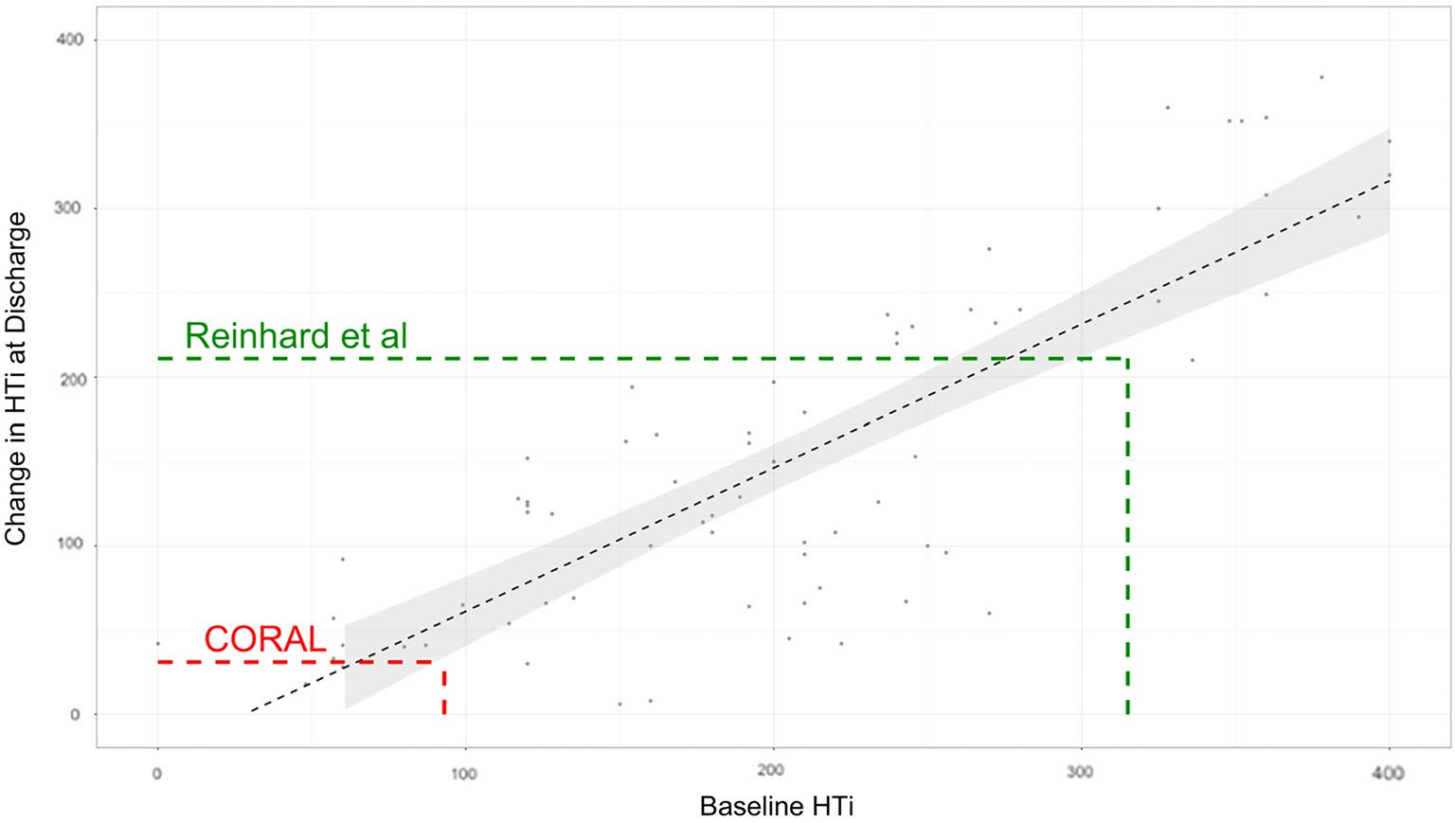
Comparing the baseline hypertension index with the impact of treatment in trials of renal artery stenting. HTi = Hypertensive Index.

The beneficial impact of treatment varied by outcome measure: 46% showing an improvement in both medication load and blood pressure, 29% showing a benefit in blood pressure with no reduction in medication load, and 9% showing no benefit in either parameter. Those with the highest Hypertensive Index prior to intervention had the best response to treatment (Fig. 2). This finding was supported in the analysis of two trials of renal artery stenting. Patients entering the CORAL trial had a mean pre-intervention HTi of 93, and gained minimal benefit from treatment. In contrast, Reinhard et al. observed significant benefit following stenting in their cohort, with a mean pre-intervention HTi of 315 (Fig. 3).

**Figure 4:**
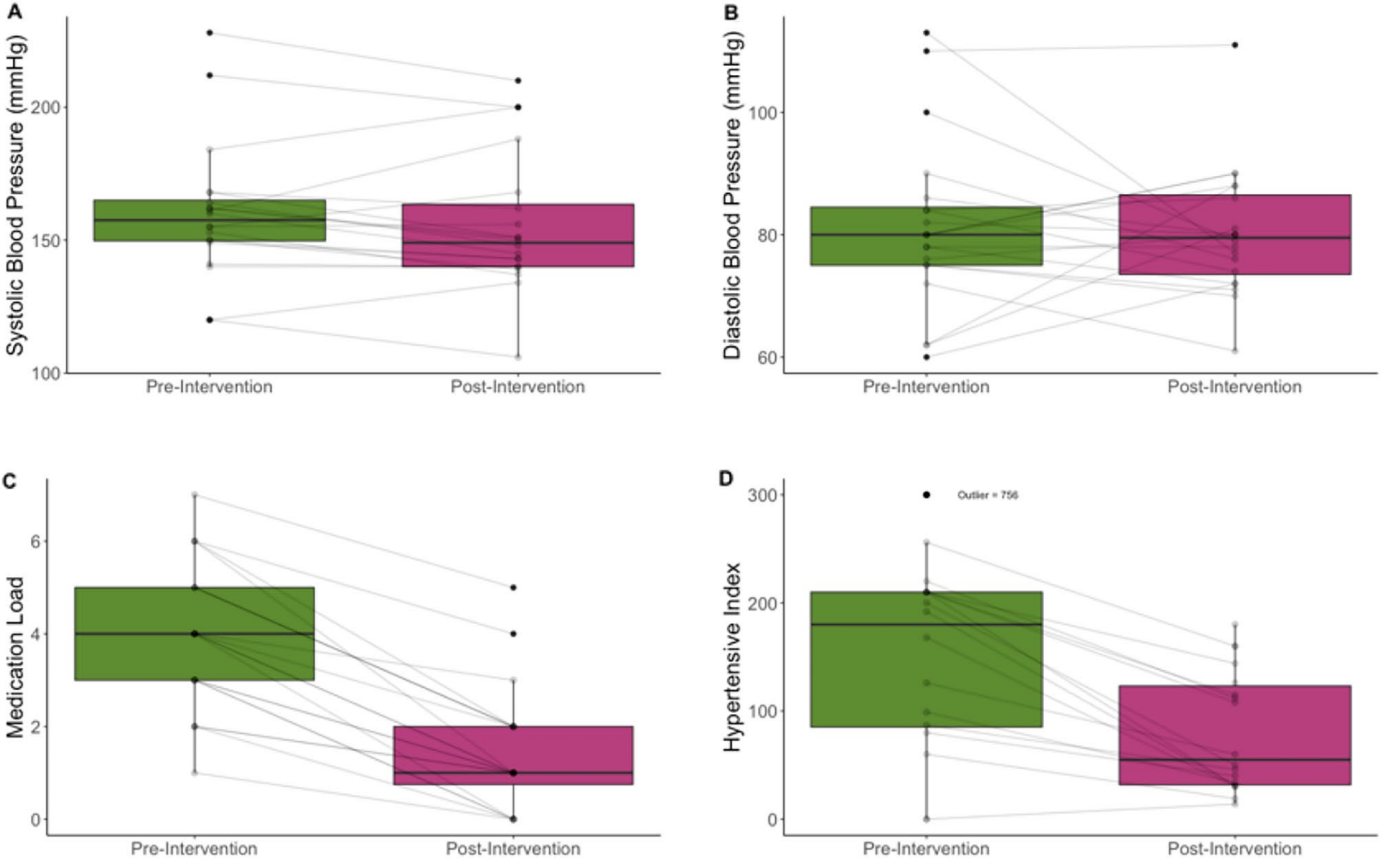
Changes in response to renal artery intervention at discharge in those without significant blood pressure reduction. Key: N = 15. (**A**) systolic blood pressure (**B**) diastolic blood pressure (**C**) number of antihypertensive medications (**D**) Hypertensive Index.

Subsets were then allocated based on the response to treatment (Table 2, Table 3). When considering the individual element of improved outcome of blood pressure, 15% had a reduction in medication without an impact on blood pressure (Group 3). Their dependence on fewer antihypertensive agents is, however, demonstrated in the reduction in their Hypertensive Index scores (Fig. 4).

**Table 2:** Changes in the Hypertensive index by response to medication and blood pressure.

	Hypertensive Index				
	Baseline		Discharge		p-value
	Median	95% ci	Median	95% ci	
Group 1 (BP ↓, Drug Rx ↓) (n = 46)	350	290	20	82	< 0.0001
Group 2 (BP ↓, Drug Rx ↔) (n = 29)	237	171	60	80	< 0.0001
Group 3 (BP ↔ , Drug Rx ↓)(n = 15)	204	190	75	64	0.0008
Group 4 (BP ↔ , Drug Rx ↔)(n = 9)	160	72	152	40	0.1

*ci* confidence interval.

**Table 3:** Blood pressure and medication values by group.

	Systolic blood pressure		Diastolic blood pressure		Medications	
	Baseline	Discharge	Baseline	Discharge	Baseline	Discharge
Group 1 (BP ↓, Drug Rx ↓) (n = 46)	199.1 (25.9)	133.7 (23.5)	96.5 (20.7)	78.5 (13.3)	3.4 (1.1)	2.5 (1.1)
*p-value*	< 0.0001		< 0.0001		< 0.0001	
Group 2 (BP ↓, Drug Rx ↔) (n = 29)	193.1 (23.9)	139.2 (21.9)	95.8 (21.7)	73.5 (13.2)	2.6 (1.4)	2.6 (1.4)
*p-value*	< 0.0001		< 0.0001		1	
Group 3 (BP ↔ , Drug Rx ↓) (n = 15)	160.1 (25.6)	155.6 (25.9)	81.3 (14.0)	80.2 (10.4)	4.0 (1.6)	1.4 (1.4)
*p-value*	0.1297		0.7169		< 0.0001	
Group 4 (BP ↔ , Drug Rx ↔) (n = 9)	167.9 (25.7)	156.7 (25.4)	81.5 (12.2)	76.3 (10.4)	2.9 (1.1)	2.9 (1.1)
*p-value*	0.07		0.106		0.3356	

Blood pressures and medication values described as mean (sd).

**Figure 5:**
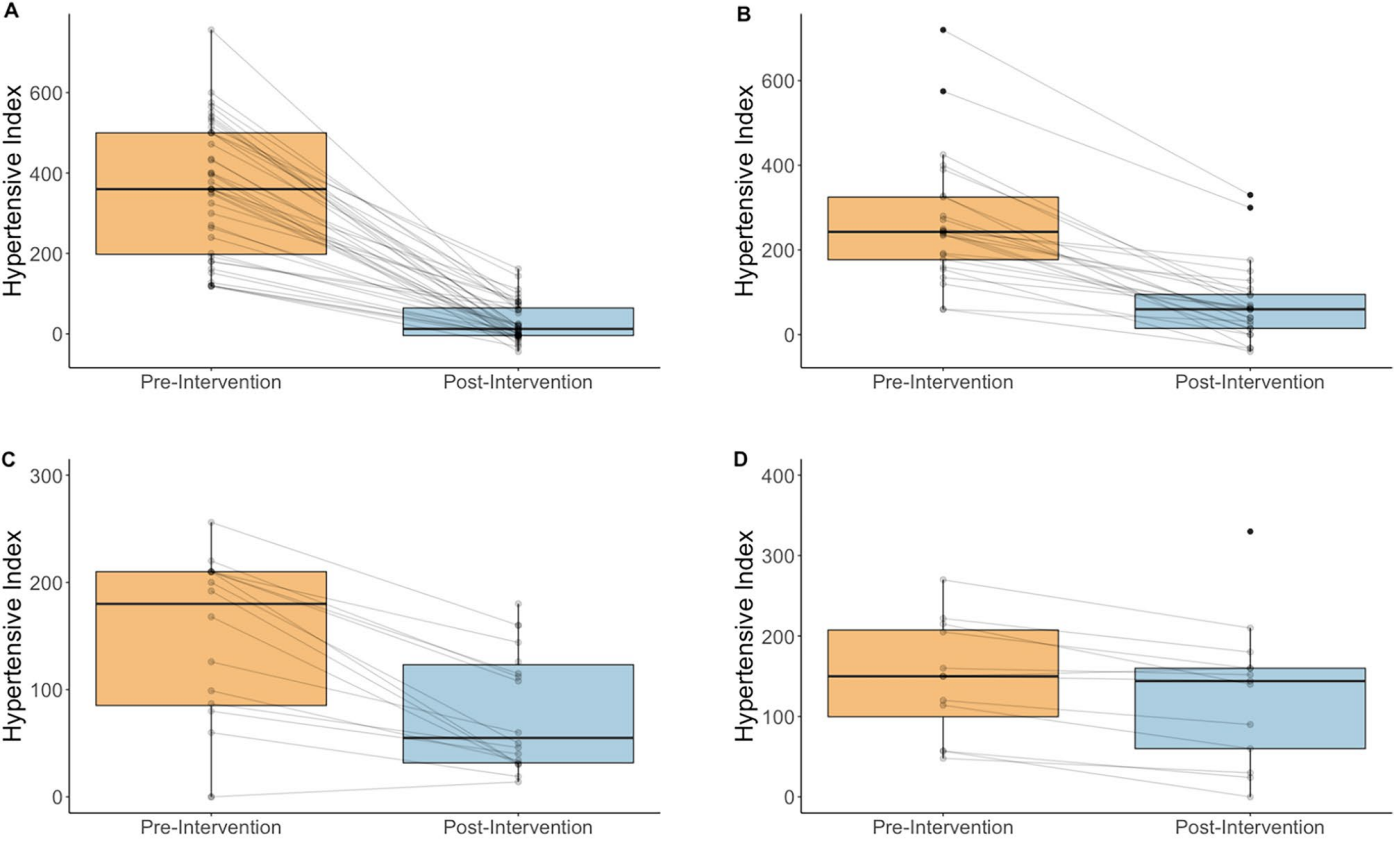
Changes in hypertensive index at discharge following renal artery intervention, by subgroup. Key: (**A**) Group 1 (**B**) Group 2 (**C**) Group 3 (**D**) Group 4.

The Hypertensive Index showed statistically significant change in all patients who achieved a significant change in blood pressure or a reduction in antihypertensive medications as a result of intervention. In those who achieved neither (Group 4), there was no significant change in the Hypertensive Index (Fig. 5). Similarly, 4 patients in whom it was not possible to fully revascularize or remove an ischaemic kidney failed to show any difference in the HTi.

### Comparison with other measures

**Figure 6:**
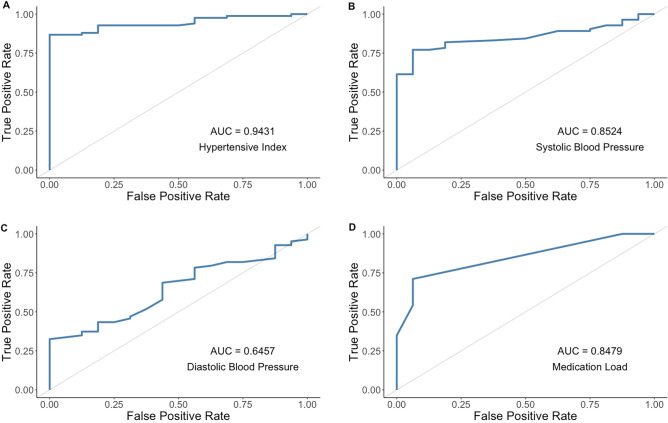
ROC analysis comparing the hypertensive index with other measures of success. AuC: area under the curve. Key: (**A**) Hypertensive Index (**B**) Systolic Blood Pressure (**C**) Diastolic Blood Pressure (**D**) Medication Load.

Receiver operating characteristic (ROC) analysis showed that the Hypertensive Index was superior to blood pressure measurements or medication load alone in identifying a successful intervention (Fig. 6).

**Figure 7:**
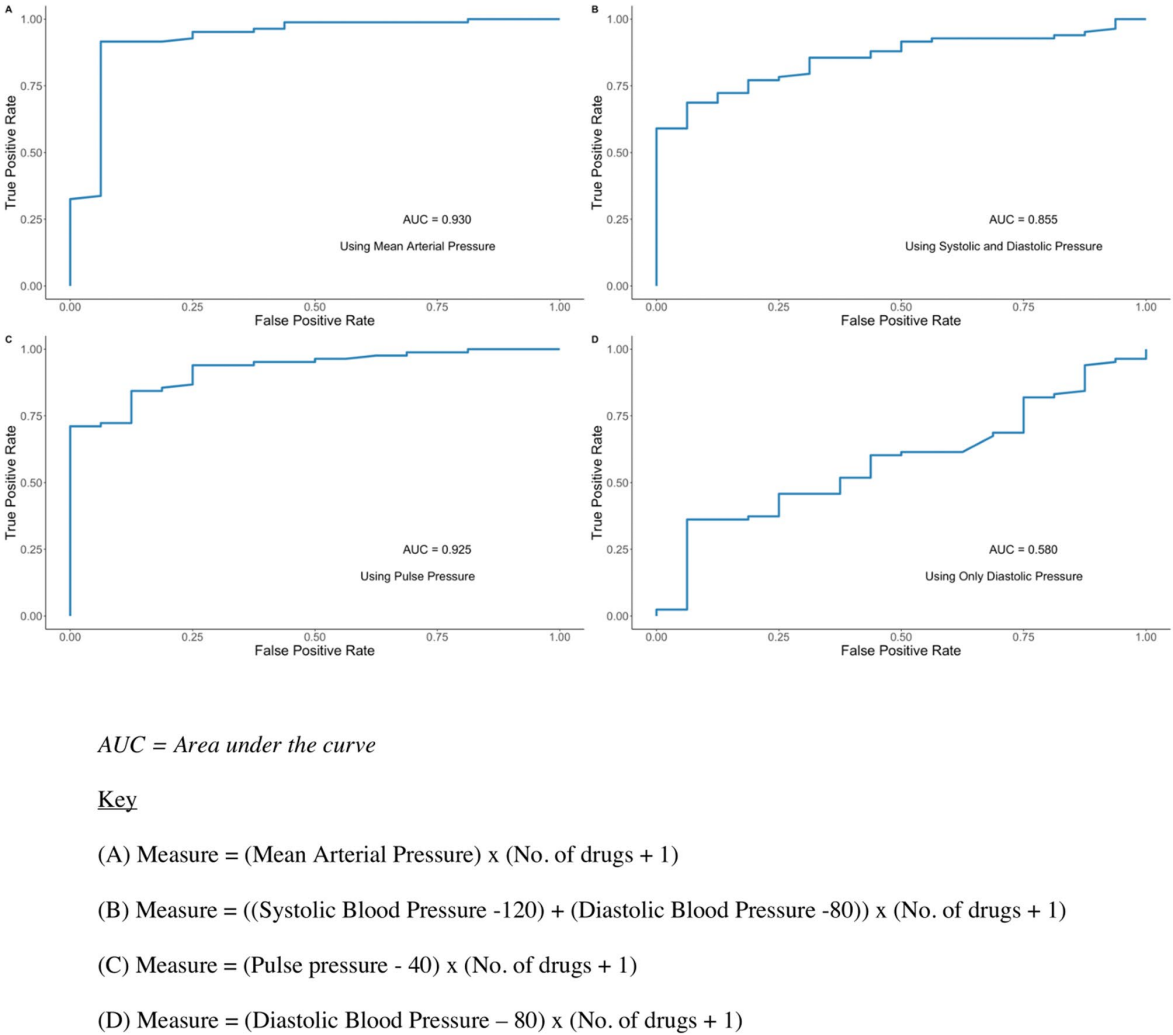
Alternative trialled methods of calculating a Hypertensive Index using measures other than systolic blood pressure alone.

The addition of alternative combinations including diastolic blood pressure, mean arterial pressure and pulse pressure failed to improve the sensitivity of the HTi in identifying beneficial change (Fig. 7).

**Figure 8:**
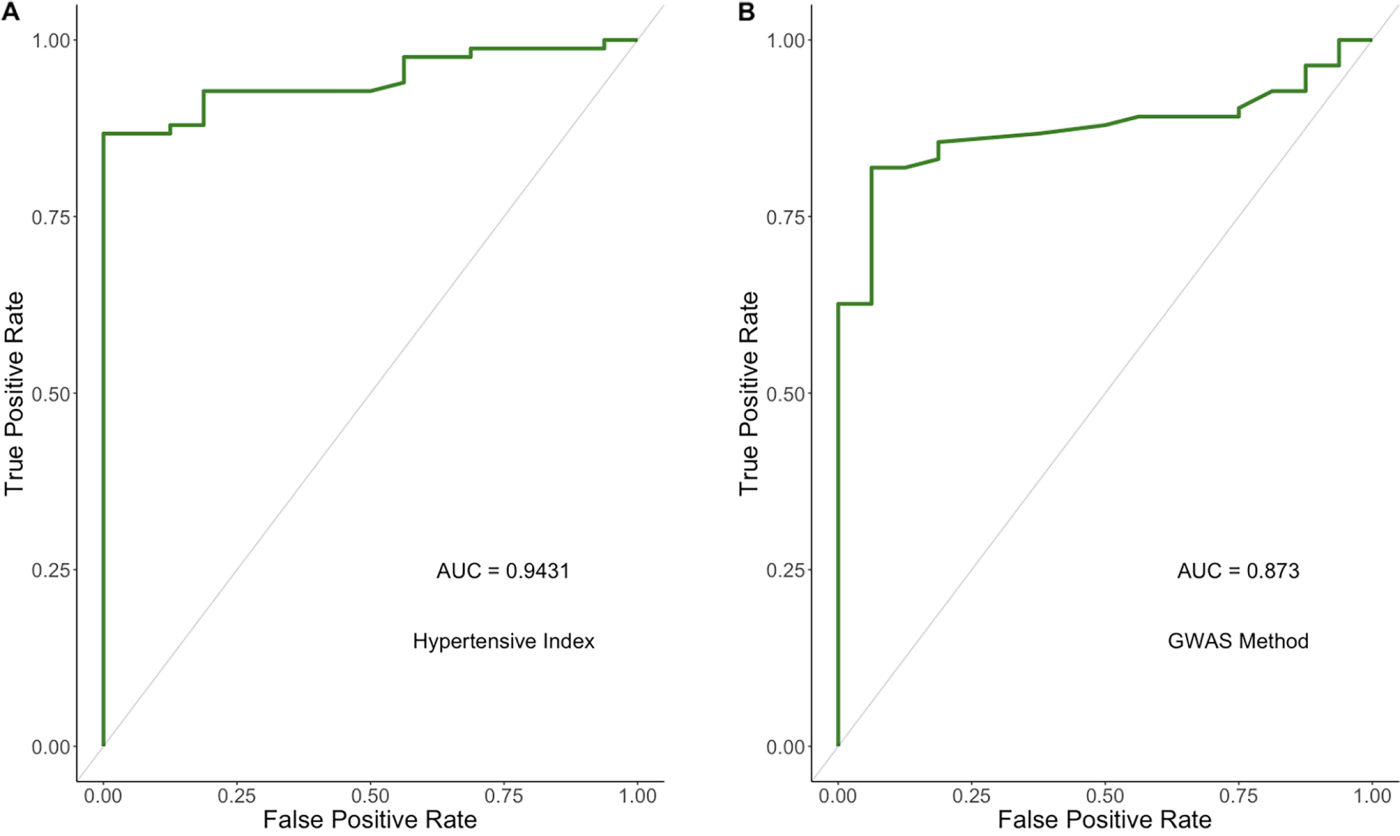
Comparing the Hypertensive Index with the method used in the Genome-wide association study on blood pressure. Key: (**A**) Hypertensive Index (**B**) GWAS Study method.

The HTi was then compared against the GWAS method of defining blood pressure^8^. Whilst the GWAS method outperformed blood pressure or medication load alone, it had a smaller area under the curve (AUC) than the Hypertensive Index (Fig. 8).

## Discussion

The aim of treating hypertension is to effectively reduce the absolute level of blood pressure. whilst minimising the drug burden in the number of medications. It is increasingly recognised that the prescription complexity itself is an additive burden that may impact on compliance. However, these twin aims of hypertensive treatment may be obscured in trials of mechanical treatment by the impact on, and reporting of, the two endpoints separately—the absolute blood pressure and the number of medications to achieve this. Measurement of either, in isolation, may lead to an underestimate of the overall benefit from intervention. Thus, a novel measure of the burden of hypertension was derived that is based on the equal contribution of both factors to an individual patient—the Hypertensive Index. Using this as an outcome measure in patients who have undergone interventions for hypertension has shown this to be an interesting measure of effective treatment that may be more holistic than either parameter taken in isolation, and a better measure of therapeutic impact.

The HTi was calculated using two parameters, both of which may be criticised. The use of an ideal baseline systolic blood pressure of 120 mmHg was based on international guidelines, meta-analysis and trials that prioritised the impact of treatment on systolic, rather than diastolic blood pressure. For example, a recent meta-analysis of all RCT of single agent antihypertensive RCT was based on the impact on systolic measurements^4^. Furthermore, the addition of alternative combinations failed to improve the sensitivity of the HTi in identifying beneficial change (Fig. 7). Given that the risk attributed to hypertension is linear and a continuous variable, using the exact amount of blood pressure in excess of an ideal may allow a more refined guide of individual risk^14^. This pattern of risk is less well recognised for other blood pressure markers of hypertension. Thus patients who have asymmetrical hypertension with differing extent of elevation of systolic to diastolic blood pressure, or who have an asymmetrical impact from treatment with one or the other being more impacted by treatment, would not be recognised using this method. However there were no patients in the small sample size used in this study who fitted this pattern, and thus this would not undermine the basis of the HTi as proposed.

The second variable—the medication load, may also be questioned. Whilst the approach used is simplistic (the number of drugs plus one), it is a practical and pragmatic measure of hypertension severity. This may require refinement if ongoing studies of lower total doses of multiple drugs or single polypharmacy pills are successful. To date however, single and additive medication prescription remains the basis of antihypertensive regimens. In addition, the effectiveness of the overall regimen is the basis of the HTi, and thus achieving better blood pressure control will outweigh an increase in the number of medications, e.g., comparing 145 mmHg on 2 tablets (HTi of 75) rather than 130 mmHg on 4 tablets (HTi of 50).

A comparison of the HTi to another method (GWAS) demonstrated a better performance. However, this would require a larger sample size to determine if this difference is clinically relevant. It is notable however, that the alternative methodology of describing ‘treated blood pressure’ was based on estimation of variance components applied to a pre-existing dataset^15^, with an arbitrary value of 10 mmHg used as an average impact on systolic blood pressure. As the HTi uses data from each individual patient—measured blood pressure and prescribed medication load, it provides a more direct, individualised, and nuanced measure of the effect of intervention on blood pressure that measures the differing impact of medication on individual patients.

There is a recognised limitation in the lower end of the HTi with an achieved systolic blood pressure of 120 leading to a score of 0, irrespective of the number of medications employed. This is due to there being no excess risk from increased blood pressure. Similarly, a blood pressure lower than 120 will achieve a negative score, which itself be a marker of risk or over-treatment.

Whilst the findings are encouraging, the study cohort is small, and would ideally be replicated in larger data series, for example using a dataset from a large well-recognised RCT. The HTi, when applied to the mean measures of blood pressure and medication load in the CORAL trial, demonstrated that the predicted impact of treatment would overall be very low—as observed in the trial^10^. Applying this measure to the individual data may be revealing. The next step would require validation, ideally by prospective use in larger randomised controlled trials of blood pressure treatment, and its utility in predicting the risk of cardiovascular events should be investigated.

In conclusion, this study describes a novel scoring system which combines excess blood pressure and medication load into a single combined score. This may be a more sensitive marker of treatment effect than assessing blood pressure measurements alone. The use of such scoring systems in future trial design may allow more accurate reporting of the effects of interventions for hypertension.

## Data Availability

The datasets generated during and/or analysed during the current study are available from the corresponding author on reasonable request.
